# **β** Cell mass expansion during puberty involves serotonin signaling and determines glucose homeostasis in adulthood

**DOI:** 10.1172/jci.insight.160854

**Published:** 2022-11-08

**Authors:** Anne-Laure Castell, Clara Goubault, Mélanie Ethier, Grace Fergusson, Caroline Tremblay, Marie Baltz, Dorothée Dal Soglio, Julien Ghislain, Vincent Poitout

**Affiliations:** 1 Montreal Diabetes Research Center, Centre de recherche du centre hospitalier de l’Université de Montréal (CRCHUM), Montreal, Quebec, Canada.; 2 Department of Medicine and; 3 Department of Pharmacology and Physiology, University of Montreal, Quebec, Canada.; 4 CHU Sainte-Justine, Montreal, Quebec, Canada.; 5 Department of Pathology and Cell Biology, University of Montreal, Montreal, Quebec, Canada.

**Keywords:** Endocrinology, Metabolism, Beta cells

## Abstract

Puberty is associated with transient insulin resistance that normally recedes at the end of puberty; however, in overweight children, insulin resistance persists, leading to an increased risk of type 2 diabetes. The mechanisms whereby pancreatic β cells adapt to pubertal insulin resistance, and how they are affected by the metabolic status, have not been investigated. Here, we show that puberty is associated with a transient increase in β cell proliferation in rats and humans of both sexes. In rats, β cell proliferation correlated with a rise in growth hormone (GH) levels. Serum from pubertal rats and humans promoted β cell proliferation, suggesting the implication of a circulating factor. In pubertal rat islets, expression of genes of the GH/serotonin (5-hydroxytryptamine [5-HT]) pathway underwent changes consistent with a proliferative effect. Inhibition of the pro-proliferative 5-HT receptor isoform HTR2B blocked the increase in β cell proliferation in pubertal islets ex vivo and in vivo. Peripubertal metabolic stress blunted β cell proliferation during puberty and led to altered glucose homeostasis later in life. This study identifies a role of GH/GH receptor/5-HT/HTR2B signaling in the control of β cell mass expansion during puberty and identifies a mechanistic link between pubertal obesity and the risk of developing type 2 diabetes.

## Introduction

Puberty is a period of considerable dynamic hormonal changes characterized by activation of the hypothalamic/pituitary/gonadal (HPG) axis with subsequent secretion of sex steroids and an increase in growth hormone (GH) release at its highest rate in life. Linked to the action of these hormones, puberty is marked by an accumulation of fat mass and a decrease in insulin sensitivity associated with hyperinsulinemia (1–5). While insulin sensitivity is restored at the end of puberty in normal-weight youth, insulin resistance persists in obese adolescents (6–8). Childhood obesity is a major risk factor for metabolic and cardiovascular complications, including type 2 diabetes (T2D) (9–11). In a longitudinal study, Reinehr et al. (12) showed that in the context of obesity, entering into puberty doubles the risk of developing metabolic complications in both males and females. Hence, puberty, much like intrauterine and early postnatal development, appears to be a critical period during which metabolic stress can lead to the development of metabolic disease later in life (13, 14). Thus, it is critical to identify the factors contributing to changes in glucose homeostasis during puberty under physiological and pathological conditions of obesity, and to understand how such factors contribute to an increased risk of T2D in young adulthood (15).

Expansion of β cell mass occurs under physiological (e.g., pregnancy) or pathological (e.g., obesity) conditions of insulin resistance, and these demand a commensurate rise in insulin output from the pancreatic β cells to maintain glucose homeostasis (16, 17). In rodents and humans, β cell mass expansion results from several mechanisms, including replication of existing β cells (18). Although limited evidence suggests that β cell replication increases in humans during puberty (19, 20), the mechanisms controlling β cell proliferation in the face of pubertal insulin resistance remain largely unexplored.

At the onset of puberty, gonadotropin-releasing hormone 1–expressing (GNRH1-expressing) neurons in the hypothalamus secrete GNRH1, which activates receptors in the anterior pituitary to produce luteinizing hormone (LH) and follicle-stimulating hormone (FSH). LH and FSH then stimulate gonadal steroid secretion of estrogens from the ovaries and testosterone from the testes. In addition to their direct endocrine actions on target tissues, sex steroids stimulate GH production from the pituitary to activate the GH/insulin-like growth factor 1 (IGF1) axis (21, 22). By binding to the GH receptor (GHR) expressed in the β cell (23), GH is implicated in β cell mass expansion perinatally and in pregnancy via autocrine/paracrine serotonin (5-hydroxytryptamine [5-HT]) signaling (24–26). During the perinatal period, GH stimulates 5-HT production in β cells by increasing levels of the 5-HT synthesizing enzyme tryptophan hydroxylase 1 (TPH1), which in turn stimulates β cell proliferation via the GPCR HTR2B (25). During pregnancy, 5-HT acts downstream of placental lactogens, which signal via the prolactin receptor to stimulate β cell proliferation (26–28). In the maternal β cell, TPH1 and HTR2B expression and 5-HT production are increased, while expression of the inhibitory isoform of the 5-HT receptor HTR1D is reduced. GHR signaling also plays critical roles in regulated insulin secretion and compensatory β cell proliferation in obese mice (29), and 5-HT acting via HTR2B and HTR3A promotes insulin secretion in rodent and human islets (30, 31). However, whether GH/5-HT signaling contributes to β cell adaptation to puberty has not been established.

The present study was designed to accomplish the following: (a) determine whether β cells undergo a wave of replication during puberty in rodents and humans, (b) investigate the role of the GH/GHR/5-HT/HTR2B signaling pathway in controlling β cell proliferation during puberty, and (c) assess whether high-fat feeding during puberty in rats compromises the β cell adaptive response and leads to abnormal glucose homeostasis in adulthood.

## Results

### Puberty in rats is associated with glucose intolerance and a decrease in insulin sensitivity in both sexes.

**Figure 1:**
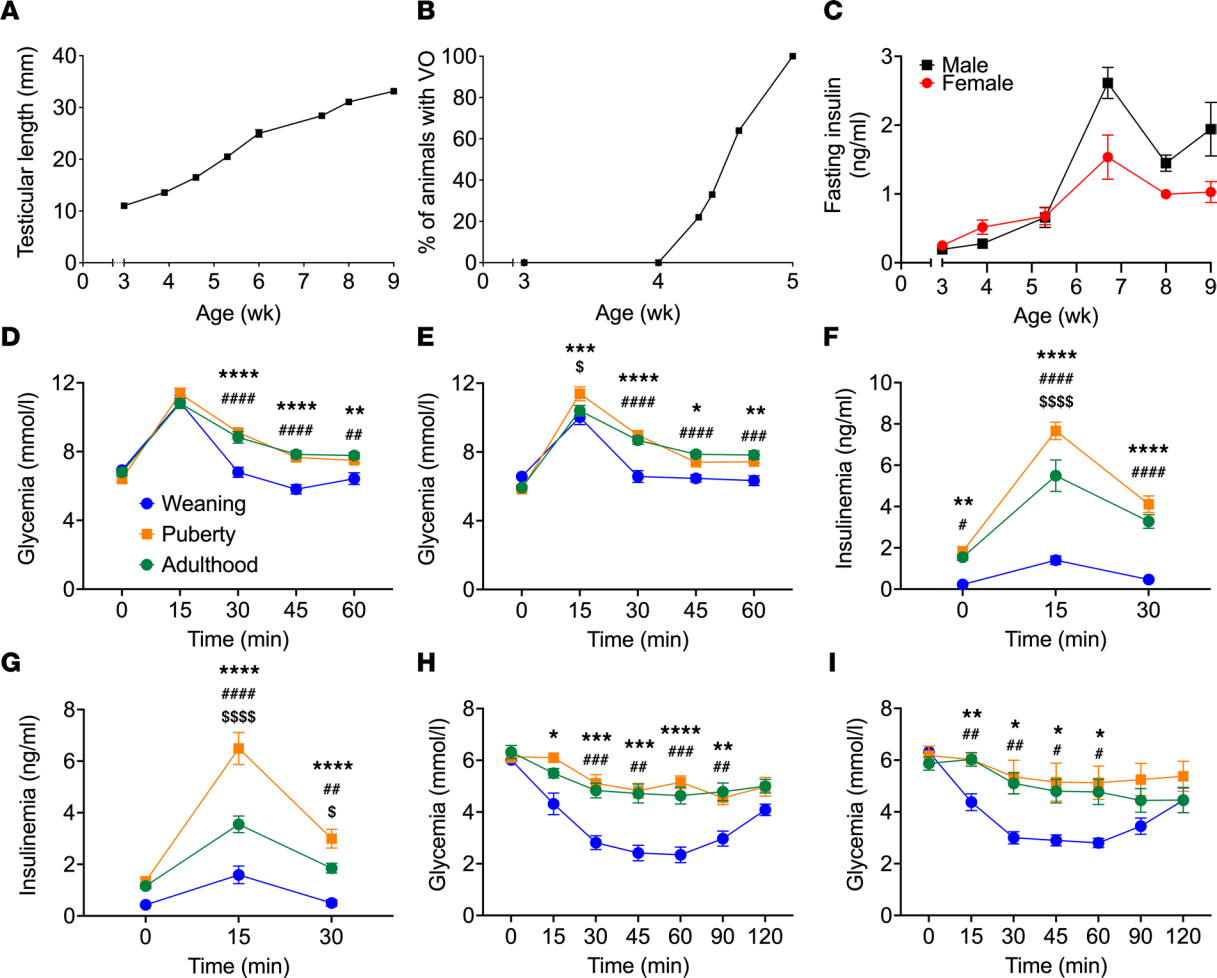
Puberty in female and male rats was associated with glucose intolerance and lower insulin sensitivity. (**A**) Right testicular length in male rats (*n* = 6). (**B**) Percentage of female rats with vaginal opening (VO) (*n* = 18). (**C**) Fasting insulin levels in male (black square) and female (red circle) rats (*n* = 6–9). (**D**–**G**) Glycemia (**D** and **E**) and insulinemia (**F** and **G**) following IPGTT (1 g/kg) in male (**D** and **F**) and female (**E** and **G**) rats at weaning (3 wk of age, blue), puberty (~6 wk of age, orange), or young adulthood (~9 wk of age, green) (*n* = 10–15). (**H** and **I**) Glycemia following IPITT in male (**H**) and female (**I**) rats at weaning (3 wk of age, blue), puberty (~6 wk of age, orange), or young adulthood (~9 wk of age, green) (*n* = 6–8). Data are expressed as mean ± SEM. *P < 0.05, **P < 0.01, ***P < 0.005, ****P < 0.001 comparing puberty and weaning groups; ^#^*P* < 0.05, ^##^*P* < 0.01, ^###^*P* < 0.005, ^####^*P* < 0.001 comparing the adult and weaning groups; and ^$^*P* < 0.05, ^$$$$^*P* < 0.001 comparing the puberty and adult groups following 2-way ANOVA (**D**–**I**) with Tukey’s multiple comparisons test.

To establish a preclinical model of β cell compensation during puberty, we characterized glucose homeostasis in male and female rats from weaning to adulthood (Figure 1 and Supplemental Figure 1; supplemental material available online with this article; https://doi.org/10.1172/jci.insight.160854DS1). Based on the clinical evaluation of the secondary sex characteristics (testicular length in male and vaginal opening in female) and changes in body weight, we defined the peripubertal period in males between 4.5 and 7.5 weeks (wk) of age and puberty onset in females beginning at 4 wk of age (Figure 1, A and B, and Supplemental Figure 1A). The occurrence of puberty earlier in female compared with male rats is consistent with findings from previous studies (32). During this period, we observed a significant increase in fasting insulinemia in both sexes (Figure 1C) with stable blood glucose levels (Supplemental Figure 1B). I.p. glucose tolerance tests (IPGTT) revealed a decrease in glucose tolerance in male (Figure 1D) and female (Figure 1E) rats at puberty that persisted at early adulthood (approximately 8–9 wk of age) in both sexes (Figure 1, D and E). Higher insulin excursions were observed during the IPGTT in male and female rats at puberty compared with weaning or adulthood (Figure 1, F and G). I.p. insulin tolerance tests (IPITT) confirmed that puberty is associated with lower insulin sensitivity in both sexes compared with weaning-stage rats (Figure 1, H and I), and this lower insulin sensitivity persisted into adulthood. Comparable levels of glucose-stimulated insulin secretion were detected in islets isolated at weaning and puberty in both sexes (Supplemental Figure 1, C and D). However, insulin secretion was higher in the presence of 8.3 mM glucose in islets isolated from adult males compared with weaning but similar at 16.7 mM glucose between all stages (Supplemental Figure 1C). In contrast, in females, glucose-stimulated insulin secretion in the presence of 16.7 mM glucose was higher in islets isolated at weaning and puberty compared with adulthood (Supplemental Figure 1D).

### β Cell proliferation increases during puberty in rats and humans.

**Figure 2:**
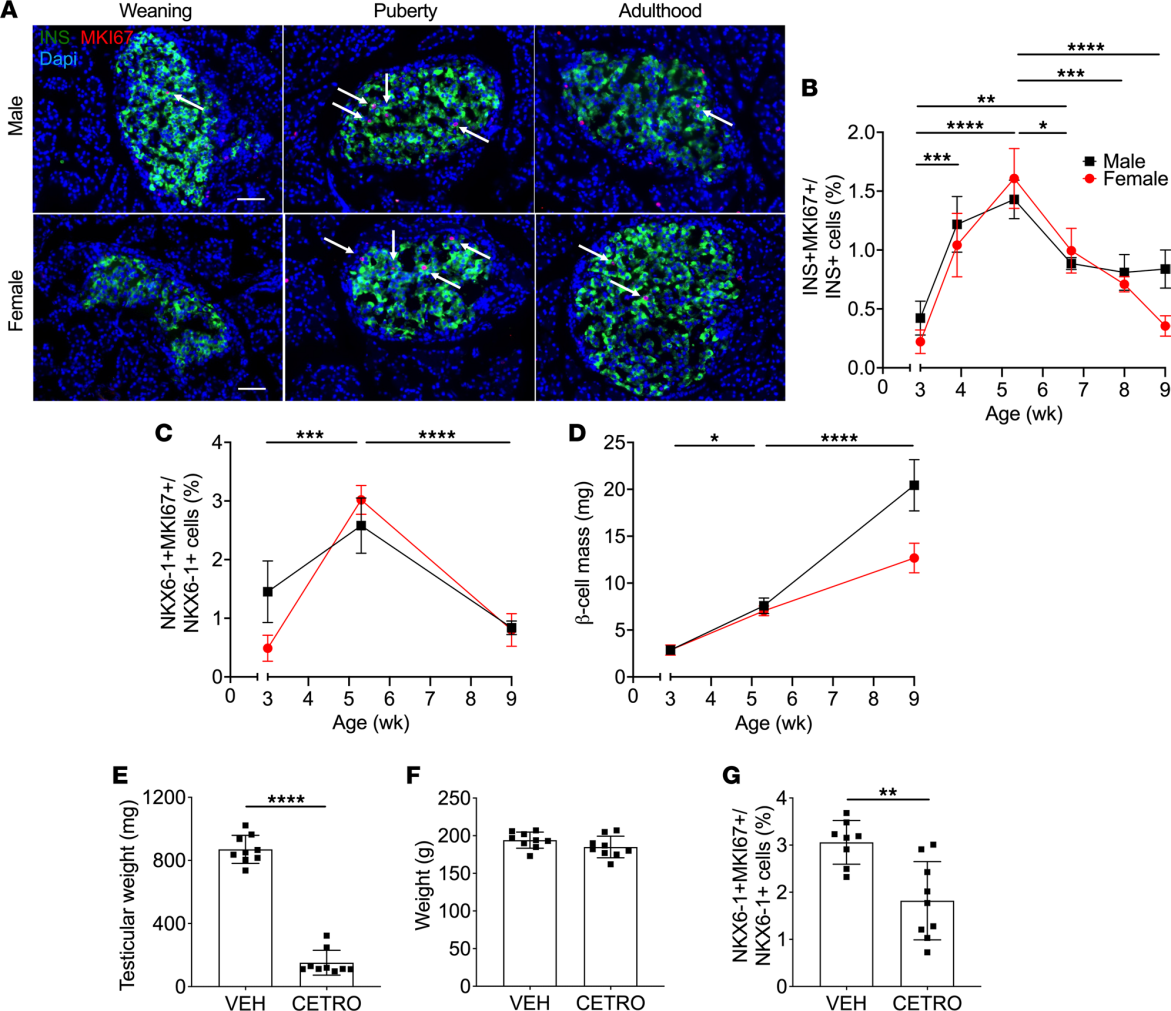
β Cell proliferation and mass were increased during puberty in rats, and β cell proliferation was blocked by GNRH1 antagonism. (**A**–**C**) β cell proliferation as assessed by immunofluorescent staining of pancreatic sections for MKI67 and insulin (INS) (**A** and **B**) or NKX6-1 (**C**) in male and female rats from 3–9 wk of age. (**A**) Representative sections showing Ins (green), MKI67 (red), and nuclei (DAPI, blue) from male (top) and female (bottom) rats at weaning (3 wk of age), puberty (~5 wk of age), or young adulthood (9 wk of age). Arrows show positive nuclei for MKI67. Scale bars: 50 μm. (**B** and **C**) β Cell proliferation as a percentage of MKI67^+^INS^+^ cells over INS^+^ cells in males (black square) (*n* = 3–5) and females (red circle) (*n* = 3–5) (**B**), or as a percentage of MKI67^+^NKX6-1^+^ cells over NKX6-1^+^ cells in males (black square) (*n* = 6–8) and females (red circle) (*n* = 6–8) (**C**). (**D**) β Cell mass in males (black square) (*n* = 5–6) and females (red circle) (*n* = 5–6). (**E**–**G**) Male rats treated with Cetrorelix (CETRO; 100 μg/d) or vehicle (VEH) from D25 to D37 (*n* = 8–9). Right testicular weight (**E**), body weight (**F**), and β cell proliferation (**G**) were assessed at D38. β Cell proliferation was measured by immunofluorescent staining of pancreatic sections for MKI67 and NKX6-1 and presented as a percentage of MKI67^+^NKX6-1^+^ cells over NKX6-1^+^ cells. Data represent individual or mean values and are expressed as mean ± SEM. *P < 0.05, **P < 0.01, ***P < 0.005, ****P < 0.001 following 1-way ANOVA with Tukey’s multiple-comparison test (**B**–**D**) or following unpaired Student’s *t* test compared with the VEH group (**E**–**G**).

To determine whether the increase in circulating insulin observed during puberty was due to a rise in β cell mass, we assessed β cell proliferation and mass by immunostaining of pancreatic sections from male and female rats sequentially from weaning to adulthood (Figure 2, A–D, and Supplemental Figure 2A). The onset of puberty in both sexes was associated with a significant and transient increase in the percentage of MKI67^+^/INS^+^ cells (Figure 2, A and B), which was confirmed by staining for NKX6-1 as a complementary β cell marker (Figure 2C and Supplemental Figure 2A). Correspondingly, β cell mass increased over time from weaning to adulthood in both sexes (Figure 2D), and this increase was associated with an overall increase in islet size in males (Supplemental Figure 2B) but not females (Supplemental Figure 2C). To demonstrate that the increase in β cell proliferation was effectively triggered by the onset of puberty, male rats were treated with the GNRH1 receptor (GNRHR) antagonist Cetrorelix (33). Cetrorelix blocked the onset of puberty, as shown by the decrease in testicular weight (Figure 2E) and testosterone levels (Supplemental Figure 2D), without changes in either body weight (Figure 2F) or IGF1 levels (Supplemental Figure 2E), and it also diminished β cell proliferation from at 5 wk of age (Figure 2G).

**Figure 3:**
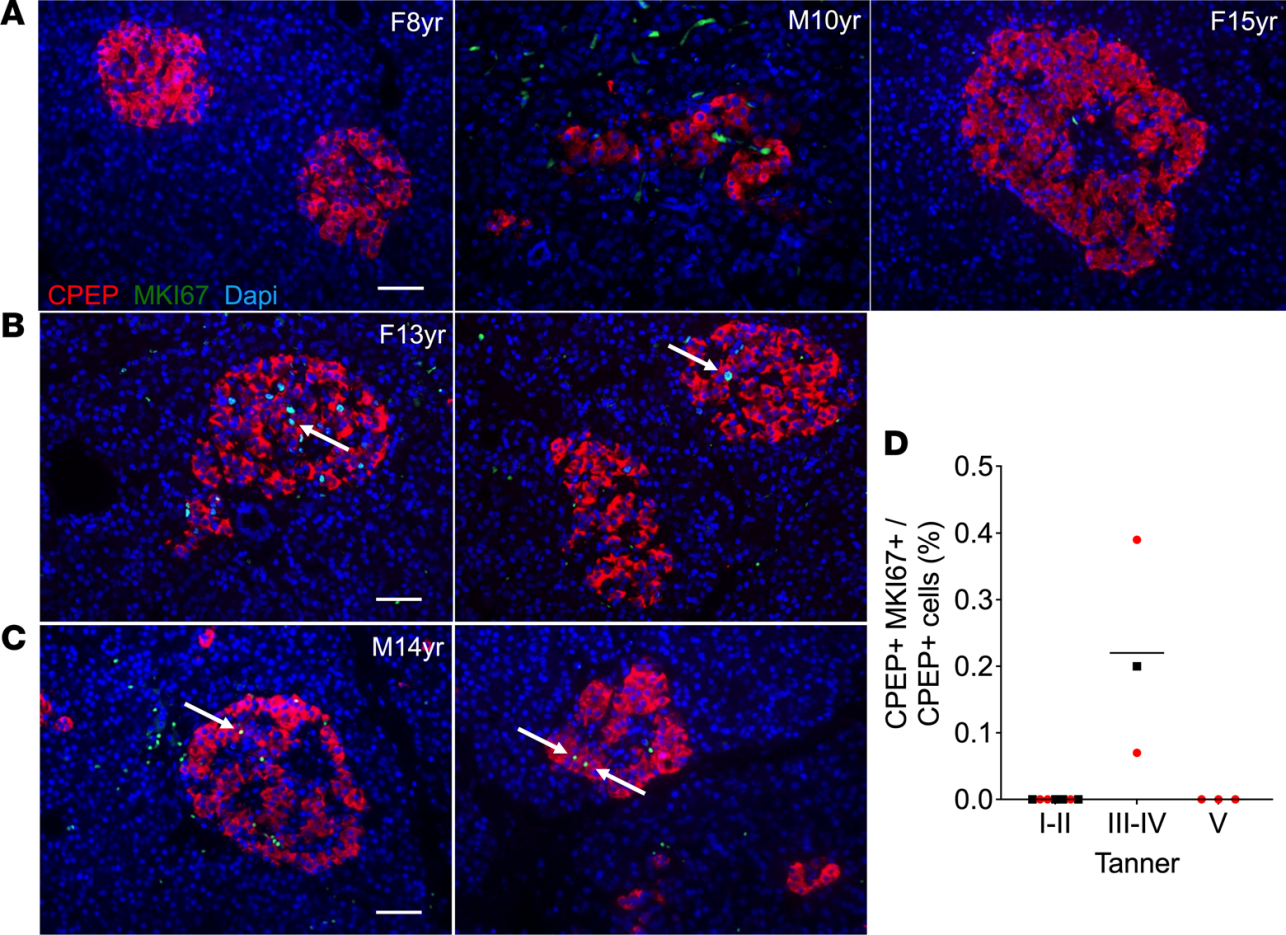
β Cell proliferation was increased during puberty in humans. β Cell proliferation in postmortem pancreatic sections of male (M) and female (F) children from 8 to 15 years old as assessed by immunofluorescent staining for MKI67 and C-peptide (CPEP). (**A**–**C**) Representative sections showing CPEP (red), MKI67 (green), and nuclei (DAPI, blue) from Tanner stages I and II (8-year-old female and 10-year-old male) and Tanner stage V (15-year-old female) (**A**), and Tanner stages III and IV (13-year-old female and 14-year-old male) (**B** and **C**), as indicated. Arrows show positive nuclei for MKI67. Scale bars: 50 μm. (**D**) β Cell proliferation presented as a percentage of MKI67^+^CPEP^+^ cells over CPEP^+^ cells grouped according to Tanner stage in males (black square) (*n* = 5) and females (red circle) (*n* = 8). Statistical analyses were not performed due to the low number of samples at each stage and sex.

In postmortem pancreatic samples from 8- to 15-year-old children (5 males and 8 females) we detected several MKI67^+^/CPEP^+^ cells in all samples at pubertal Tanner stages III and IV (corresponding to the peak of puberty; ref. 34) both in males (Figure 3, C and D) and females (Figure 3, B and D), but there were none in prepuberty and onset-of-puberty (Tanner stages I and II), or postpuberty (Tanner stage V) donors (Figure 3, A and D).

### Pubertal serum stimulates β cell proliferation in rat and human islets.

**Figure 4:**
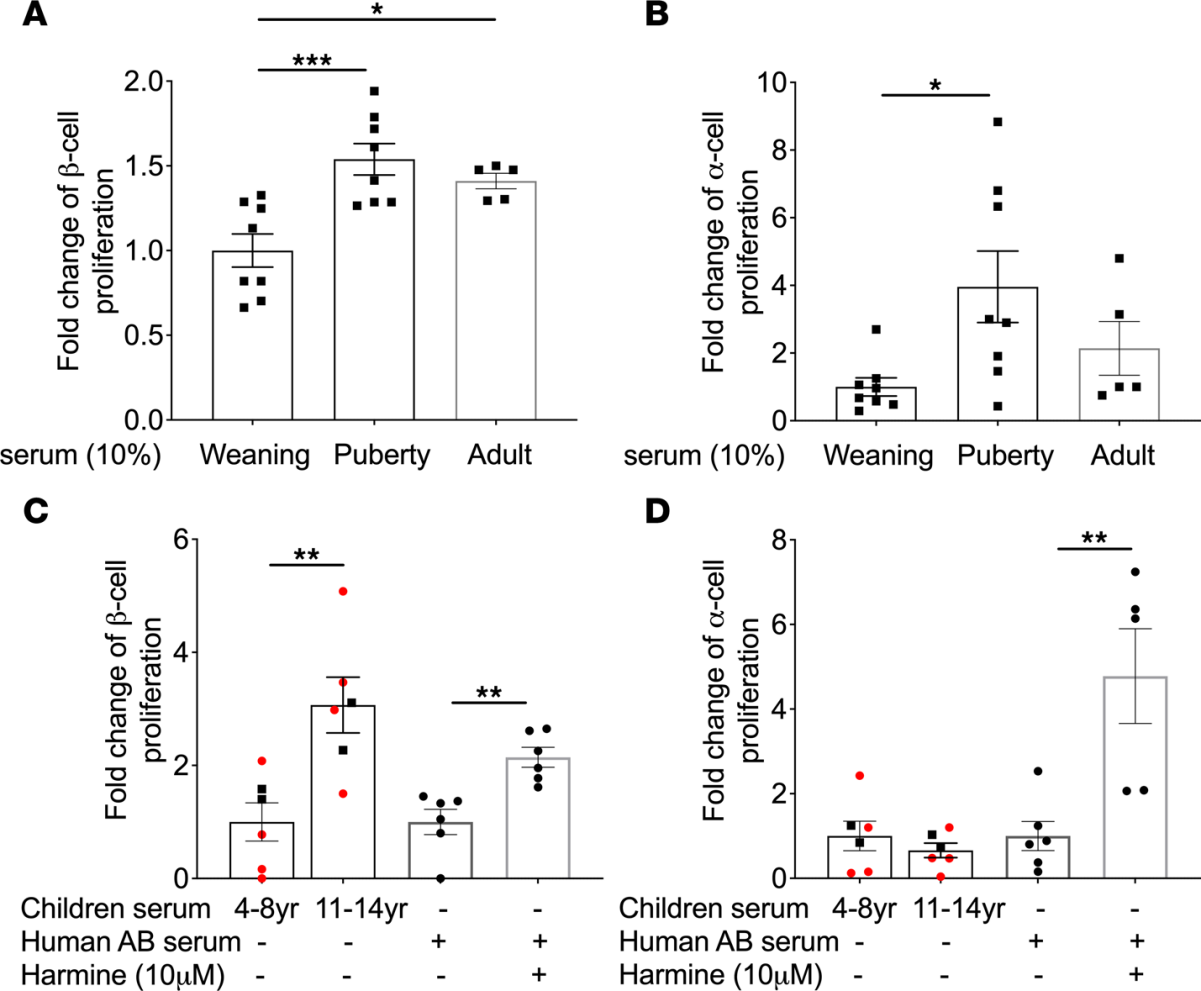
Pubertal but not prepubertal serum stimulated β cell proliferation in isolated rat and human islets. (**A** and **B**) Male rat islets were isolated at puberty were exposed to prepubertal (Weaning), pubertal (Puberty), or adult rat serum for 72 hours, and β and α cell proliferation was assessed by flow cytometry following staining for EdU and C-peptide (CPEP) or glucagon (GCG), respectively. Both β (**A**) and α (**B**) cell proliferation were presented as the fold-change of the percentage of EdU^+^CPEP^+^ or EdU^+^GCG^+^ cells over CPEP^+^ or GCG^+^ cells, respectively, over the control condition (Weaning serum) (*n* = 5–8). (**C** and **D**) Adult human islets were exposed to sex-matched prepubertal (4–8 years) or pubertal (11–14 years) human male (black square) or female (red circle) serum (*n* = 6) or to Harmine (10 μM) in the presence of human AB serum (*n* = 6) for 72 hours, and β and α cell proliferation were assessed by flow cytometry following staining for EdU and insulin (INS) or GCG, respectively. Both β (**C**) and α (**D**) cell proliferation were presented as the fold-change of the percentage of EdU^+^INS^+^ or EdU^+^GCG^+^ cells over INS^+^ or GCG^+^ cells, respectively, over the control condition (prepubertal serum and AB serum, respectively). Data represent individual values and are expressed as mean ± SEM. *P < 0.05, **P < 0.01, ***P < 0.005 following 1-way ANOVA with Tukey’s multiple-comparison test (**A** and **B**) or following unpaired Student’s *t* test (**C** and **D**).

To investigate whether β cell proliferation at puberty onset is mediated by a circulating factor, we exposed rat islets to decomplemented weaning, pubertal, or adult rat serum for 72 hours and measured islet cell proliferation by flow cytometry. Pubertal serum significantly increased α and β cell proliferation in islets isolated at puberty compared with serum collected at weaning (Figure 4, A and B). In islets isolated at weaning and young adulthood, pubertal rat serum promoted β but not α cell proliferation (Supplemental Figure 3, A–D). Although adult serum increased β but not α cell proliferation in pubertal islets (Figure 4, A and B), no increase in proliferation was detected in either α or β cells in islets isolated at weaning (Supplemental Figure 3, A and B). Accordingly, decomplemented human serum from children > 11 years old, but not children < 8 years old, increased β cell proliferation in sex-matched adult human islets (Figure 4C). Interestingly, unlike in rat islets (Figure 4B), human α cells did not proliferate in response to pubertal serum (Figure 4D). To confirm that β and α cells were responsive to proliferative signals, islets were exposed to the DYRK1A inhibitor harmine, a potent α and β cell mitogen (35). As expected, harmine increased α and β cell proliferation in all human donor islet batches (Figure 4, C and D).

These data suggest that β cell proliferation during puberty in rodents and humans is triggered by a circulating factor.

### HTR2B signaling is implicated in β cell proliferation during puberty.

**Figure 5:**
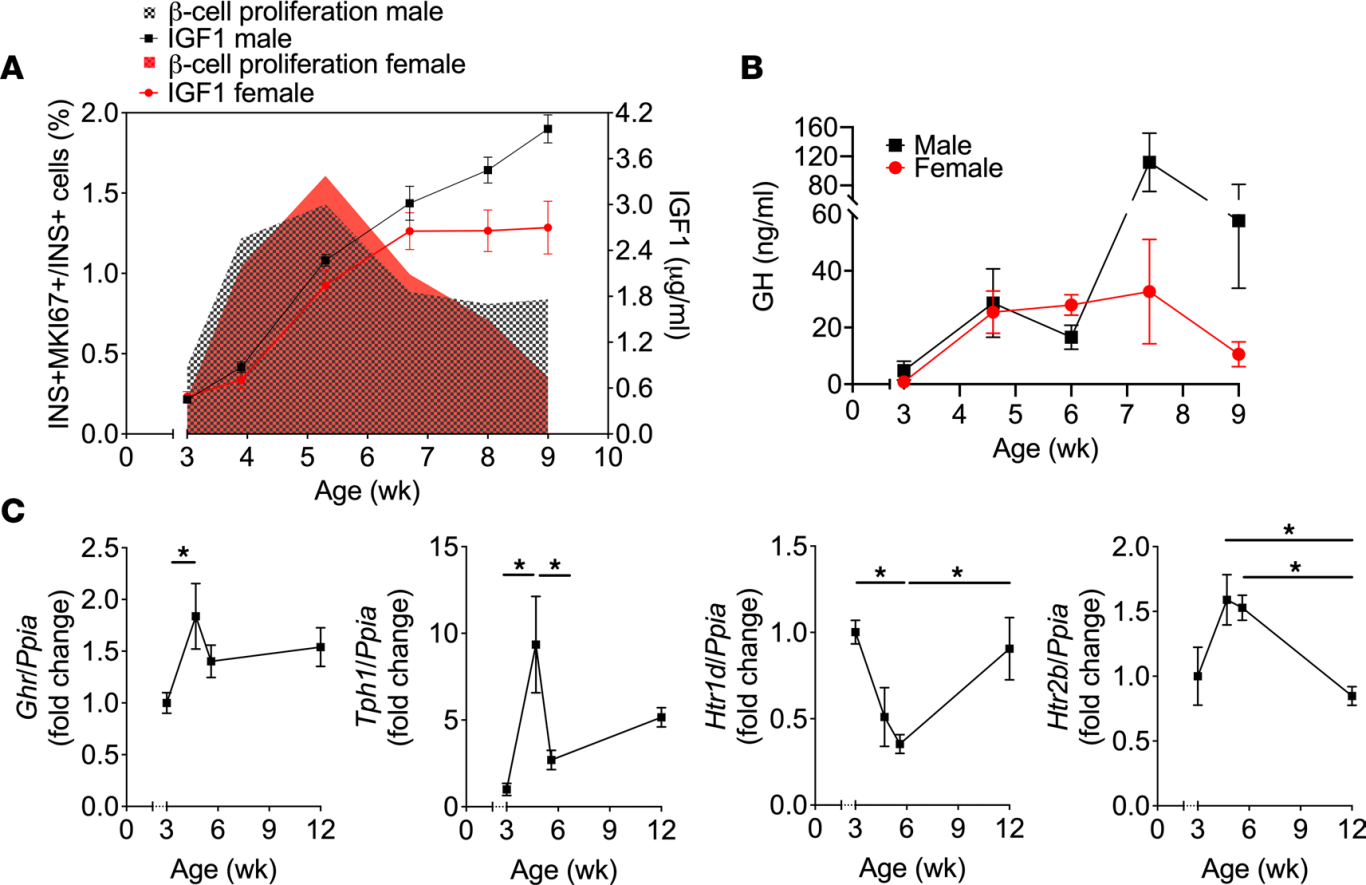
β Cell proliferation during puberty correlated with plasma IGF1/GH levels and GH/5-HT pathway gene expression in islets. (**A**) Plasma IGF1 levels and β cell proliferation (from Figure 2B) in female (red) and male (black) rats from 3–9 wk of age (*n* = 4–7). (**B**) Plasma GH levels in female (red) and male (black) rats from 3–9 wk of age (*n* = 4-5). (**C**) *Ghr*, *Tph1*, *Htr1d*, and *Htr2b* mRNA levels in rat islets isolated at different ages as indicated (*n* = 4–5). mRNA was quantified by reverse transcription PCR (RT-PCR) and normalized to cyclophilin (*Ppia*). Data are presented as the fold change over the prepubertal time point (3 wk of age). Data are expressed as mean ± SEM. *P < 0.05 following 1-way ANOVA with Tukey’s multiple-comparison test (**C**).

**Figure 6:**
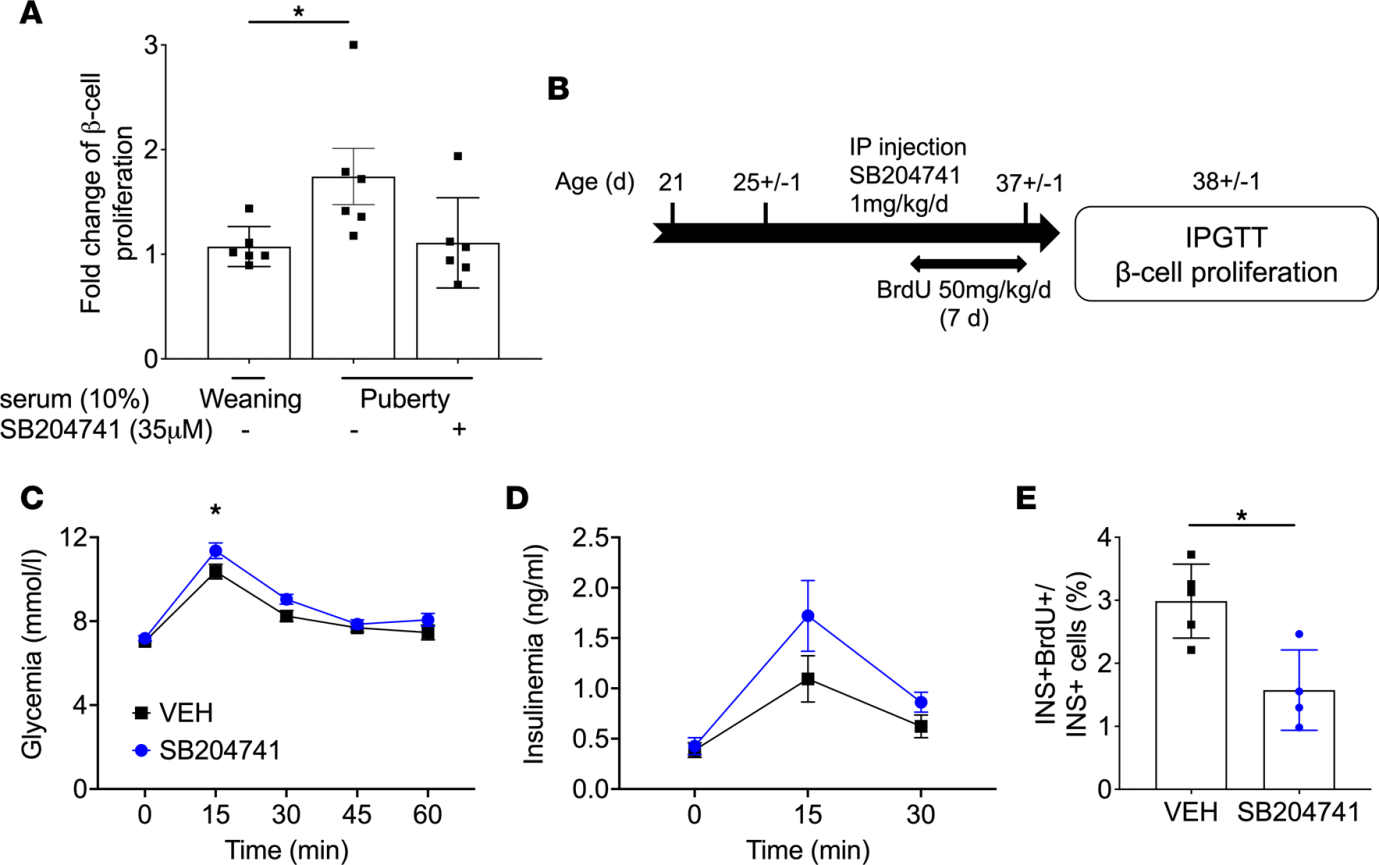
β Cell proliferation during puberty was blocked by HTR2B antagonism in rat islets ex vivo and in vivo. (**A**) Male rat islets isolated at puberty were exposed to 10% weaning (3-wk-old) or pubertal (~5-wk-old) rat serum in the presence of SB204741 (35 μM) for 72 hours, and β cell proliferation was assessed by flow cytometry following staining for EdU and insulin (INS) and presented as the fold-change of the percentage of EdU^+^INS^+^ over INS^+^ cells over the control condition (Weaning serum) (*n* = 6–7). (**B**) Male rats were exposed to SB204741 (1 mg/kg/d) or vehicle (VEH) from D25 to D37 and BrdU from D30 to D37, after which IPGTT (1 g/kg) were performed and β cell proliferation was assessed by immunofluorescent staining of pancreatic sections for BrdU and INS. (**C** and **D**) Glycemia (**C**) and insulinemia (**D**) during the IPGTT in the SB204741-injected group (blue) and the control group (VEH, black) (*n* = 11 per group). (**E**) β Cell proliferation as a percentage of BrdU^+^INS^+^ over INS^+^ cells (*n* = 4–5). Data are expressed as mean ± SEM. *P < 0.05 following 1-way (**A**) or 2-way (**C** and **D**) ANOVA with Tukey’s multiple-comparison test or following unpaired Student’s *t* test (**E**).

GH levels increase during puberty in humans (22), and GHR signaling stimulates β cell proliferation via autocrine/paracrine 5-HT/HTR2B signaling (24–26). Hence, we asked whether this pathway mediates β cell proliferation during puberty in rats. First, in line with studies in humans, we detected an increase in circulating IGF1 and GH levels in both sexes during puberty, concomitant with the rise in β cell proliferation (Figure 5, A and B). Second, a membrane-filtered pubertal serum fraction ≥ 3 kDa containing GH stimulated β cell proliferation, whereas the fraction ≤ 3 kDa depleted in GH was inactive (Supplemental Figure 4, A and B). Third, β cell proliferation increased, albeit mildly, following exposure to recombinant rat GH (Supplemental Figure 4C). Fourth, mRNA expression of *Ghr*, *Tph1*, and the stimulatory 5-HT receptor isoform *Htr2b* was increased in islets at the onset of puberty, whereas that of the inhibitory 5-HT receptor isoform *Htr1d* was diminished (Figure 5C). Fifth, the selective HTR2B antagonist SB204741 (36) blocked pubertal serum–induced β cell proliferation in isolated rat islets (Figure 6A). Finally, to investigate the contribution of HTR2B signaling in vivo, we administered SB204741 to male rats from postnatal day 25 (D25) to D37 and assessed glucose tolerance and β cell proliferation at D38 (Figure 6B). Body weight was similar in both SB204741- and vehicle-treated groups (Supplemental Figure 4D). During the IPGTT, animals treated with SB204741 showed higher glycemia at 15 minutes (Figure 6C) and higher area under the glucose curve during the first 30 minutes of the test (Supplemental Figure 4E), suggesting mild glucose intolerance. Insulinemia was not significantly different between the groups (Figure 6D and Supplemental Figure 4F), yet the number of BrdU^+^ β cells was lower in the SB204741-injected group at D38 (Figure 6E).

These data suggest that HTR2B signaling is implicated in β cell proliferation during puberty.

### Insulin, estrogen, and androgen receptor signaling are not involved in pubertal serum–induced β cell proliferation.

Since insulin levels were elevated at puberty (Figure 1C), and insulin receptor signaling is necessary for β cell compensation to insulin resistance (37), we asked whether insulin might mediate the increase in β cell proliferation in response to pubertal serum. However, insulin in decomplemented pubertal serum was undetectable, and the insulin receptor antagonist, S961 (38), did not diminish pubertal serum–induced β cell proliferation (Supplemental Figure 5, A and B).

Since the GNRHR antagonist Cetrorelix diminished pubertal β cell proliferation (Figure 2G) and sex hormone receptor signaling controls β cell function and mass (39, 40), we evaluated the role of sex steroids in pubertal serum–induced β cell proliferation. Pubertal islets exposure to estrogen and androgen receptor antagonists — ICI 182,780 and flutamide, respectively (39, 41) — had no effect on β cell proliferation induced by pubertal serum (Supplemental Figure 5C).

### Peripubertal high-fat diet is associated with glucose intolerance and reduced β cell mass in adulthood.

**Figure 7:**
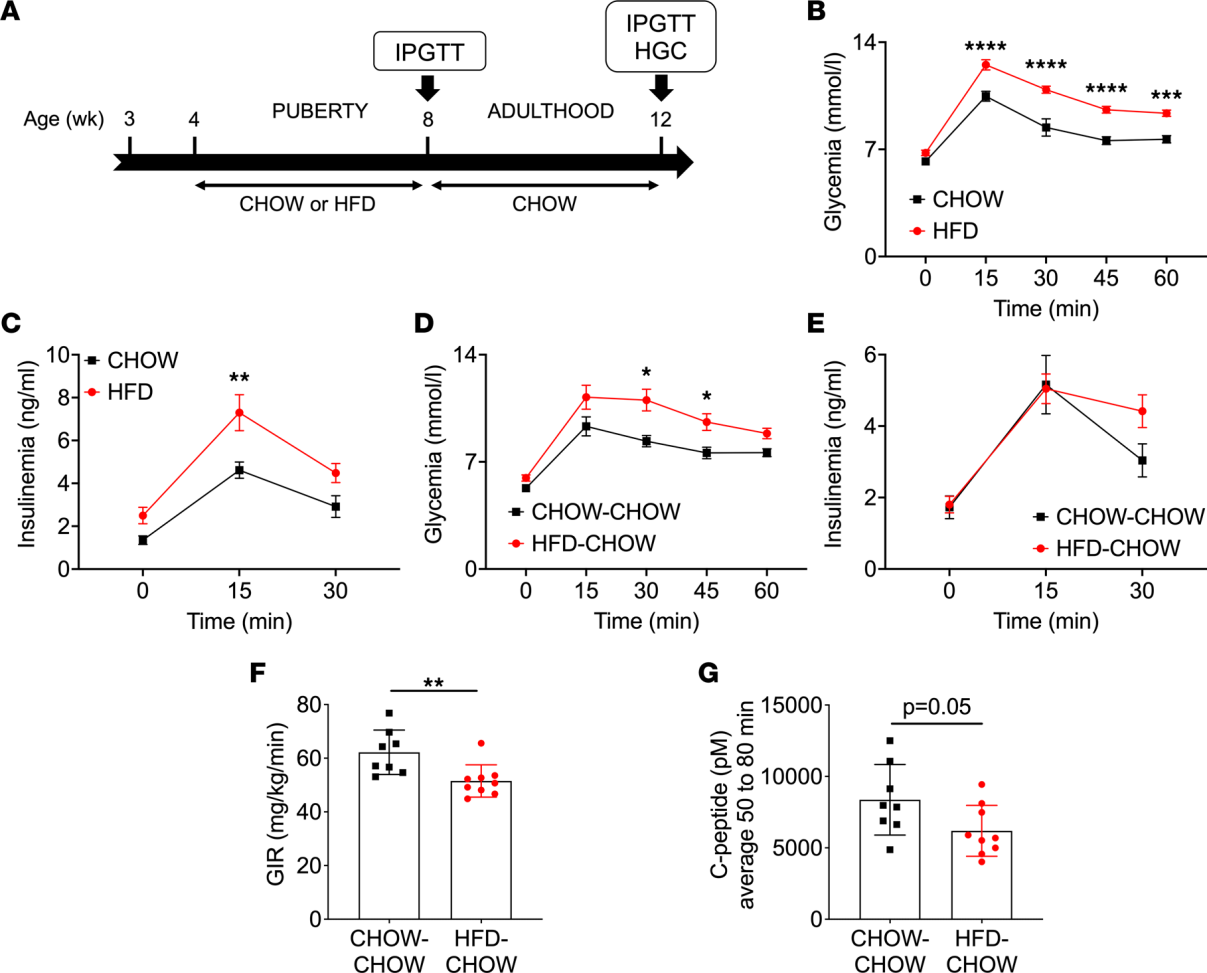
HFD during puberty led to glucose intolerance in adulthood. (**A**) IPGTT and HGC were performed on male rats fed a HFD (HFD, red) or a chow diet (CHOW, black) from 4 to 8 wk of age followed by a switch to a chow diet for both groups (HFD-CHOW, red; CHOW-CHOW, black) until 12 wk of age. (**B**–**E**) Glycemia (**B** and **D**) and insulinemia (**C** and **E**) during the IPGTT (1 g/kg) performed at 8 (*n* = 7–9) (**B** and **C**) and 12 (*n* = 6-9) (**D** and **E**) wk of age. (**F** and **G**) Glucose infusion rate (GIR) (**F**) and C-peptide levels (**G**) during the HGC performed at 12 wk of age (*n* = 8–9). Data are expressed as mean ± SEM. *P < 0.05, **P < 0.01, ***P < 0.005, ****P < 0.001 following 2-way ANOVA with Sidak’s multiple-comparison test (**B**–**E**) or unpaired Student’s *t* test (**F** and **G**).

To assess the impact of peripubertal metabolic stress on glucose homeostasis, male rats were exposed to high-fat diet (HFD) from 4 to 8 wk of age followed by normal chow diet until 12 wk of age (HFD-CHOW), or they were exposed to chow diet throughout (CHOW-CHOW) (Figure 7A). Body weight and cumulative food intake increased in the HFD-CHOW group compared with controls beginning at 8 wk of age (Supplemental Figure 6, A and B). IPGTT performed at 8 wk of age revealed glucose intolerance with higher insulinemia in the HFD-fed group compared with controls (Figure 7, B and C). Interestingly, despite the switch to chow diet at 8 wk, the animals that had been fed HFD from wk 4 to 8 were still glucose intolerant at 12 wk of age (Figure 7D), without an increase in insulin levels during the test (Figure 7E), suggesting defective insulin secretion. To further explore a potential β cell secretory defect, we performed hyperglycemic clamps (HGC) on HFD-CHOW and CHOW-CHOW groups at 12 wk of age. During the steady state of the clamp (50 to 80 minutes), blood glucose levels were in the target range in both groups (Supplemental Figure 6C). The glucose infusion rate (GIR) was lower in the HFD-CHOW group (Figure 7F), indicative of insulin resistance, and C-peptide levels trended lower (Figure 7G), consistent with defective insulin secretion.

**Figure 8:**
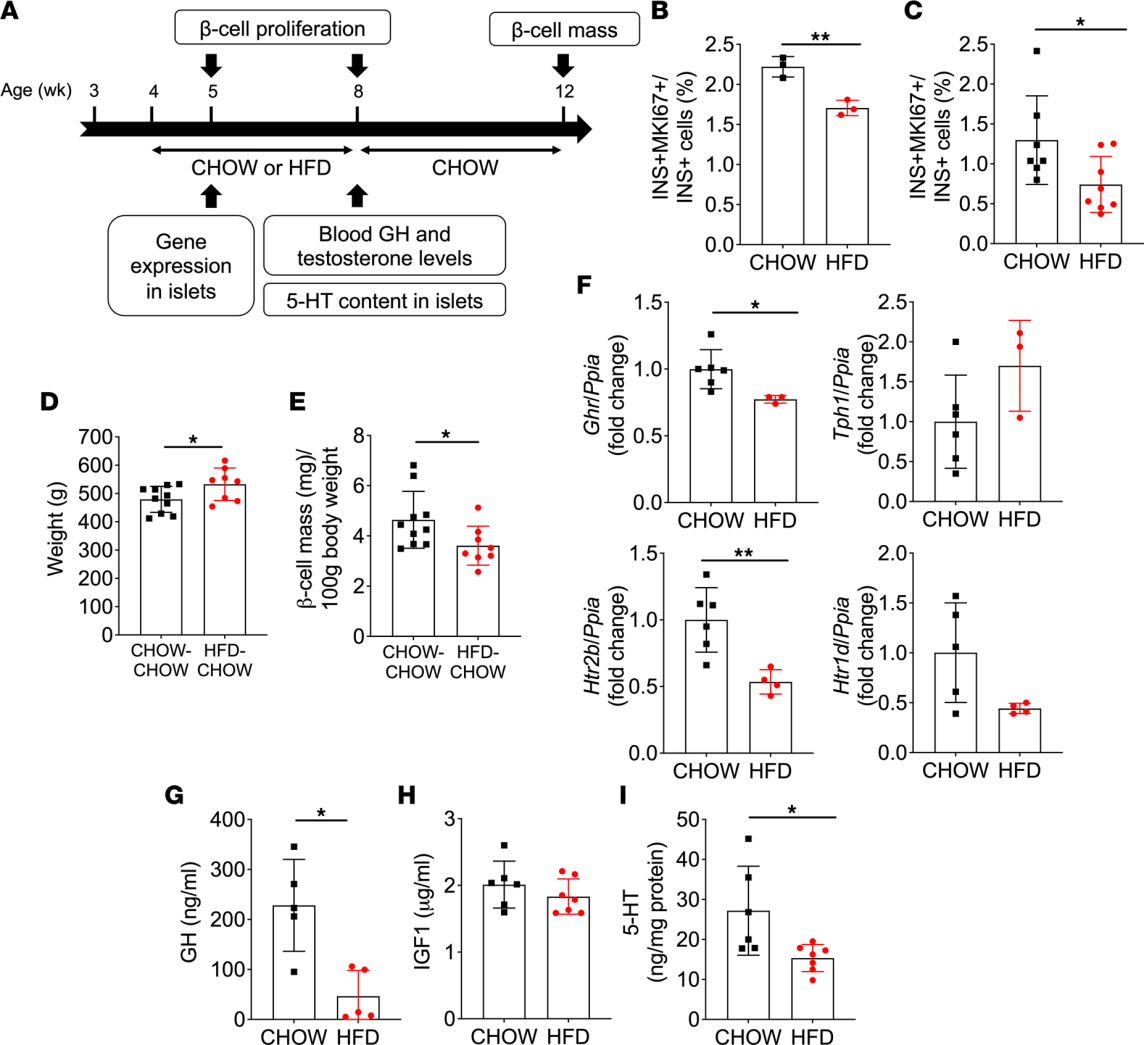
HFD during puberty mitigated β cell proliferation and GH/5-HT pathway hormone levels and gene expression, and it reduced β cell mass in adulthood. (**A**) β Cell proliferation and mass, plasma hormone and islet gene expression, and 5-HT content were assessed in male rats fed a HFD (HFD, red) or a chow diet (CHOW, black) from 4 to 8 wk of age followed by a switch to a chow diet for both groups (HFD-CHOW, red; CHOW-CHOW, black) until 12 wk of age. (**B** and **C**) β Cell proliferation was assessed at 5 (**B**) (*n* = 3) and 8 (**C**) (*n* = 7–8) wk of age by immunofluorescent staining of pancreatic sections for MKI67 and insulin (INS) and presented as the percentage of MKI67^+^INS^+^ cells over INS^+^ cells. (**D** and **E**) Body weight (**D**) and β cell mass per 100 g of body weight (**E**) at 12 wk of age (*n* = 8–10). (**F**) *Ghr*, *Tph1*, *Htr1d*, and *Htr2b* mRNA levels in islets isolated from rats at 5 wk of age (*n* = 3–6). mRNA was quantified by RT-PCR and normalized to cyclophilin (*Ppia*). Data are presented as the fold change over the CHOW diet group. (**G**–**I**) Plasma GH (**G**) and IGF1 (**H**) levels and islet 5-HT content normalized to total protein (**I**) in rats at 8 wk of age (*n* = 6-7). Data represented individual values and are expressed as mean ± SEM. *P < 0.05, **P < 0.01 following unpaired Student’s *t* test as compared with the control CHOW or CHOW-CHOW group.

To assess whether HFD during puberty impacts β cell mass expansion, we measured β cell proliferation and mass in HFD-CHOW and CHOW-CHOW groups (Figure 8A). β Cell proliferation was decreased at 5 wk (Figure 8B and Supplemental Figure 6D) and 8 wk (Figure 8C and Supplemental Figure 6E) of age in HFD-fed animals compared with the CHOW-fed group. Consequently, β cell mass normalized to body weight was reduced at 12 wk of age in the HFD-CHOW group (Figure 8, D and E).

Overall, these data indicate that pubertal HFD blunts the normal β cell proliferative response and leads to glucose intolerance and lower β cell mass in adulthood.

### Peripubertal HFD dampens GH/5-HT/HTR2B signaling in islets.

Given the role of HTR2B in pubertal β cell proliferation shown in Figure 6, we tested whether this pathway was affected by HFD (Figure 8). One wk after initiation of HFD, *Ghr* and *Htr2b* mRNA expression was decreased compared with controls, while *Tph1* and *Htr1d* were similar between groups (Figure 8F). Circulating GH levels were markedly lower after 4 wk of HFD (Figure 8G), while IGF1 levels were unchanged (Figure 8H). Accordingly, 5-HT concentrations in islets were lower in the HFD group (Figure 8I).

## Discussion

The objectives of this study were to identify the mechanisms of β cell adaptation to insulin resistance during puberty and to ascertain whether this process is perturbed by metabolic stress. We showed that puberty is associated with a transient increase in β cell proliferation mediated by a circulating factor in rats and humans of both sexes. Mechanistically, pubertal islets undergo a phenotypic switch in the expression of genes of the GH/5-HT signaling pathway that favors β cell proliferation, and inhibition of HTR2B signaling blocks the β cell response ex vivo and in vivo. Finally, peripubertal HFD blunts 5-HT signaling and the physiological rise in β cell proliferation, leading to impaired β cell mass and glucose homeostasis later in life.

We found that puberty in rats of both sexes was associated with decreased glucose tolerance and insulin sensitivity. Despite increased plasma insulin levels in response to an oral glucose load at puberty, insulin secretion in islets ex vivo was not elevated at puberty compared with weaning islets in either sex, suggesting that puberty is not associated with an intrinsic increase in β cell secretory capacity. Adult male islets were slightly more responsive to glucose than islets from weaning rats, which is in line with previous findings in male mice (42). In contrast, adult female islets exhibited diminished insulin secretion relative to islets at weaning or puberty. This was unexpected, as previous studies suggest that glucose-stimulated insulin secretion is elevated in adult female compared with male Wistar rat islets (43).

The increase in β cell proliferation detected at puberty in rats is consistent with a previous study in neonatal male Sprague Dawley rats describing an increase in β cell mass at D24 and D31 associated with an increase in β cell proliferation (44). In human samples, we detected proliferating β cells at midpuberty (Tanner stages III and IV). Accordingly, Lam et al. (20) observed an increase in islet endocrine cell proliferation in adolescents and young adults, although the proliferating islet cells did not express β cell markers, contrary to our observations. Interestingly, the increase in β cell proliferation we detected in human samples mirrors the peak of physiological insulin resistance that also occurs at Tanner stages III and IV and returns to prepubertal levels at Tanner stage V (1, 3, 4, 45). However, limited donor information precluded precise characterization of puberty by Tanner staging. Additional donors will be required to confirm the timing of pubertal β cell proliferation. Overall, the data indicate that β cells undergo a wave of proliferation in response to pubertal insulin resistance in rodents and humans.

Our observation that pubertal serum from rats and humans stimulates β cell proliferation demonstrates the involvement of a circulating factor. Importantly, the increase in β cell proliferation preceded the peak in plasma insulin levels in both sexes in rats. Furthermore, decomplemented serum was completely devoid of insulin, and insulin receptor signaling did not contribute to pubertal serum–induced β cell proliferation. These data exclude the possibility that β cell replication is directly stimulated by circulating insulin.

The reduction in β cell proliferation following administration of the GNRHR antagonist Cetrorelix in rats supports a role of the HPG axis in β cell mass expansion during puberty. Gonadotrophins released downstream of GNRH1 stimulate the secretion of testosterone and estrogens, which, in turn, trigger GH secretion from the pituitary. A possible role for sex hormones in β cell adaptation to puberty is suggested by the observation that castration of young adult male rats leads to a reduction in β cell mass, which is reversed by testosterone administration (46). Accordingly, testosterone and its metabolites dihydrotestosterone and estrogen acting via the androgen and estrogen receptors, respectively, are established regulators of β cell function and mass in males (39, 40). However, our attempts to demonstrate a role of the androgen and estrogen receptors in pubertal serum–induced β cell proliferation were unsuccessful.

GH/IGF1 are other potential candidate circulating factors that could trigger β cell proliferation in puberty. Indeed, circulating levels of IGF1, a surrogate for GH, increased concomitantly with β cell proliferation during puberty in rats. Similarly in humans, GH/IGF1 secretion are at their highest levels during puberty (47). Furthermore, GH causes insulin resistance (48–50) and pubertal insulin resistance positively correlates with GH and IGF1 levels (51, 52). Importantly, both GH (53–56) and IGF1 (57) increase β cell proliferation, while whole body and β cell–specific *Ghr* deficiency in mice are associated with a decrease in β cell proliferation and mass (25, 29, 58, 59), and β cell–specific insulin and IGF1 receptor–double KO mice show decreased β cell function and mass (60). We found that, in serum fractionation studies, only the GH-containing fraction (greater 3 kDa) retained the ability to stimulate β cell proliferation. Furthermore, GH increased β cell proliferation in pubertal islets ex vivo. Overall, our data suggest a role for GH in promoting β cell proliferation in puberty that remains to be formally confirmed by identification of the circulating factor mediating this effect.

At the islet level, we observed changes in mRNA expression similar to the pattern of expression of maternal islets during pregnancy (26) and suggestive of a phenotype consistent with 5-HT–induced proliferation — namely, increased *Tph1* and *Htr2b* and decreased *Htr1d*. Accordingly, the HTR2B antagonist SB204741 blocked the increase in β cell proliferation in isolated rat islets in response to pubertal rat serum and abrogated pubertal β cell proliferation in vivo in rats. Hence, similar to the perinatal period (25) and pregnancy (26), 5-HT signaling controls β cell proliferation during puberty and likely contributes to adult β cell mass. This model predicts that 5-HT is synthesized in β cells in response to GHR activation, acts in an autocrine/paracrine fashion, and is, therefore, entirely consistent with the possibility that GH is the circulating factor mediating this response.

Peripubertal HFD in male rats blunted the β cell proliferative response and reduced β cell mass in adulthood despite a 4-wk “wash-out” period, during which the animals received a chow diet. The decrease in β cell mass was associated with impaired glucose tolerance and insulin secretion. In agreement with our findings, Holtrup et al. (61) found that mice fed a HFD during the peripubertal period displayed glucose intolerance and insulin resistance in adulthood. Glucose homeostasis was also more severely impaired in rats fed a HFD before adulthood compared with those receiving HFD only during adulthood (62). In contrast, in a recent study, Glavas et al. (63) did not detect impaired glucose homeostasis or changes in β cell mass when mice were fed a HFD exclusively during the peripubertal period, perhaps due to lower fat content of the diet compared with the present study. Nevertheless, increased diabetes incidence was observed when HFD was initiated in mice at preweaning and peripubertal compared with postpubertal stages (63), consistent with a more severe outcome following high-fat feeding early in life. Interestingly, resistance to metabolic impairment in mice fed a HFD after weaning is associated with higher circulating IGF1 levels and lean mass (64). In contrast, although IGF1 levels were unchanged by HFD in our studies, we observed that pubertal HFD reduced plasma GH levels, consistent with a reduction in GH secretion, without changes in IGF1 levels, in humans under metabolic stress (65, 66). Pubertal HFD also reduced *Ghr* gene expression in islets and decreased islet 5-HT content and *Htr2b* gene expression. 5-HT contributes to β cell compensation to metabolic stress, as defects in insulin secretion and glucose tolerance are observed in β cell–specific *Tph1* and 5-HT receptor *Htr3a*-KO mice under HFD (30). Taken together with our data supporting a role of 5-HT in β cell proliferation at puberty, we propose that pubertal HFD leads to reduced 5-HT signaling with resulting impairment of β cell mass in adulthood. Hence, much like the perinatal period (25), pubertal β cell replication likely contributes to adult β cell mass; hence. perturbations during this period could have detrimental effects on glucose homeostasis in young adulthood.

Because of the rising prevalence of childhood obesity, prediabetes and T2D have become increasingly common in youth (67, 68). Puberty is a critical window for the establishment of metabolic health such that physiological changes during this period influence diabetes risk (12, 14). In particular, pubertal insulin resistance is exacerbated in obese adolescents and does not return to prepubertal values (6, 7). However, adolescent obesity is also associated with reduced β cell function, which may augment diabetes risk (8, 69, 70). Hence, much like during development or early postnatal life, the pubertal pancreatic islet may be a key target for metabolic programming that affects risk of metabolic disease later in life (13, 60, 71).

In conclusion, this study provides evidence for a role of GH/GHR/5-HT/HTR2B signaling during puberty in the control of β cell proliferation and mass, and it sheds light on the molecular pathways linking puberty and obesity to the risk of developing T2D later in life.

## Methods

### Reagents.

The GNRHR antagonist Cetrorelix was from Cayman Chemicals (catalog 23910-10), the selective HTR2B receptor antagonist SB204741 and the estrogen receptor antagonist ICI 182,780 were from Tocris (catalogs 1372/10 and 1047/1, respectively). Recombinant rat GH protein (rGH) was from Abcam (catalog ab68388). BrdU, the androgen receptor antagonist flutamide, and the DYRK1A inhibitor harmine were from Sigma-Aldrich (catalogs B5002, F9397, and 286044, respectively). The insulin receptor antagonist S961 was from Phoenix Pharmaceuticals (catalog 05186). The Amicon Ultra-0.5 Centrifugal Filter Units were from MilliporeSigma (catalog UFC500324). Fatty acid–free BSA was obtained from Equitech-Bio.

### Animals.

A Wistar rat (Charles River Laboratories) colony was maintained in-house. Animals were housed under controlled temperature on a 12-hour light-dark cycle with free access to water and fed ad libitum with normal chow diet (CHOW; catalog 2018 Teklad Global 18% protein rodent diet; 58% carbohydrate, 24% protein, and 18% fat on a caloric basis; Harlan Teklad) or HFD (catalog D12492; 60% fat, 20% protein, and 20% carbohydrate on a caloric basis; Research Diets Inc.). The animals received HFD or CHOW diet from 4 to 8 wk of age, and they then received CHOW diet until 12 wk of age. Body weight, food intake, and fed blood glucose were monitored weekly during the HFD study. Clinical parameters (right testicular length in males using Vernier caliper; weight and vaginal opening in females), metabolic tests, β cell proliferation, and mass were assessed at different time points from weaning to adulthood. Plasma insulin, C-peptide, testosterone, GH, and IGF1 were measured by ELISA (Alpco Diagnostics; catalogs 80-INSRT-E01, 80-CPTRT-E01, 55-TESMS-E01, 22-GHOMS-E01, and 22-IG1MS-E01, respectively).

### In vivo injections.

Male rats were injected i.p. from D25 ± 1 day to D37 ± 1 day with Cetrorelix (100 μg/day in 0.4 mL of 0.9% of NaCl), SB204741 (1 mg/kg/day in PEG400; catalog 25322-68-3; MilliporeSigma), or vehicle. At the end of the treatment, body and testicular weight, plasma testosterone, and IGF1 levels and β cell proliferation in pancreatic sections were measured. BrdU (50 mg/kg/day) was injected i.p. during the last 7 days of treatment. IPGTT and β cell proliferation were assessed at D38 ± 1 day.

### Metabolic tests.

IPGTT were performed on 4- to 5-hour–fasted rats by measuring tail blood glucose (glucometer Accu-Chek, Roche) at t = 0, 15, 30, 45, and 60 minutes and measuring plasma insulin levels at t = 0, 15, and 30 min after i.p. dextrose administration (1 g/kg).

IPITT were performed on 4- to 5-hour–fasted rats by measuring tail blood glucose at t = 0, 15, 30, 45, 60, 90, and 120 minutes after i.p. insulin administration (Humulin-R 100U/mL; 0.5U/kg; Lilly).

One-step HGC were performed in conscious, ad libitum–fed animals as described (72). Briefly, a 20% dextrose solution (Baxter) was infused via a jugular catheter. Rats initially received a 90-second bolus (140 mg/kg/min), and then the GIR was adjusted to maintain blood glucose between 15.5 and 17.7 mmol/L for 80 minutes. Blood samples were collected from an arterial catheter to measure glucose, plasma insulin, and C-peptide.

### Measurement of β cell proliferation and mass in pancreatic sections.

Pancreata were fixed for 4 hours in 4% paraformaldehyde (PFA) and cryoprotected overnight in 30% sucrose. Tissue was then embedded in OCT compound, frozen, sectioned at 8 μm, and mounted on Superfrost Plus slides (Invitrogen). Antigen retrieval was performed using sodium citrate buffer (pH 6). β Cell proliferation was measured by immunofluorescent staining for MKI67 and insulin (INS) or NKX6-1, or BrdU and INS, as described (73). Primary antibodies and dilutions are listed in Supplemental Table 1. Secondary antibodies were from Jackson ImmunoResearch. Images were acquired with a fluorescence microscope (Zeiss). β Cell proliferation was expressed as percentage of double-positive MKI67^+^ and INS^+^ or MKI67^+^ and NKX6-1^+^ cells over the total INS^+^ or NKX6-1^+^ cells, respectively, and double-positive BrdU^+^ and INS^+^ over the total INS^+^ cells. At least 1,500 β cells were manually counted per condition in 1–2 sections. The experimenter was blind to group assignments. β Cell mass and islet size were measured on paraffin sections (4 sections/pancreas at 60 μm intervals) prepared as described (74) using an anti-insulin antibody (Supplemental Table 1).

### Immunofluorescent staining of human pancreatic sections.

Formalin fixed, paraffin embedded sections were deparaffinized and rehydrated. Antigen retrieval was performed using sodium citrate buffer (pH 6). β Cell proliferation was measured by immunofluorescent staining for MKI67 and C-peptide and expressed as a percentage of double-positive MKI67^+^ and CPEP^+^ cells over the total CPEP^+^ cells as described above for rats. Primary antibodies and dilutions are listed in Supplemental Table 1. At least 1,500 β cells were manually counted per condition in 1 section. Samples were grouped according to Tanner stage. When the Tanner stage was unknown, age was used to define the pubertal stage (34).

### Rat and human serum.

Blood was collected from male rats following abdominal aorta puncture at weaning (D21) or during puberty (D37–D39) and centrifuged at 1,100*g* for 10 minutes at 4°C in BD Vacutainer SST tubes (VWR, catalog 367985). Prepubertal (4- to 8- year-old) or pubertal (11- to 14-year-old) sera from boys and girls were obtained from Innovative Research Inc. All sera were decomplemented by heating at 56°C ± 2°C during 30 minutes prior to use.

### Rat islet isolation.

Peripubertal (D37 ± 1 day) rat islets were isolated from male rats by collagenase digestion and dextran density gradient centrifugation as described previously (75) and allowed to recover overnight in RPMI-1640 supplemented with 10% (v/v) FBS (Invitrogen), 100 U/mL penicillin/streptomycin (Multicell Wisent Inc.) and 11.1 mM glucose prior to use.

### Human islets.

Upon reception, human islets were maintained in cGMP Prodo Islet Media (Standard; Prodo Laboratories) with 5% (v/v) human AB serum and 1% (v/v) glutamine/glutathione mixture prior to use.

### Rat and human islet culture.

Batches of 200 peripubertal male rat islets (D37 ± 1 day) were cultured in RPMI-1640 in the presence of 7.5 mM glucose for 4 hours prior to the addition of rat serum (10% v/v) for an additional 72 hours. Adult human islets were cultured in Prodo media (5.8 mM glucose) with decomplemented sex-matched human serum (10% v/v) or with human AB serum (5% v/v) for 72 hours. Harmine (10 μM); SB204741 (35 μM); rGH (1 μg/mL); S961 (100 nM); ICI 182,780 (10 nM); and flutamide (10 nM) were used and EdU (10 μM) was added throughout. Media were replaced daily.

### β Cell proliferation in isolated islets.

Following treatment, islets were dissociated into single cells with accutase (1 μL/islet; Innovative Cell Technologies Inc.) for 10 minutes at 37°C, and β cell proliferation was assessed by flow cytometry as described previously (76). Dead cells were labeled using the LIVE/DEAD Aqua (405 nm) (BD Bioscience). EdU was detected using Click-iT Plus EdU Alexa Fluor 488. Immunostaining was performed according to the manufacturer’s instructions (Thermo Fisher Scientific). Fluorophore-coupled primary antibodies and dilutions are listed in Supplemental Table 1. Flow cytometry was carried out using an LSRIIB flow cytometer with FACSDiva software (BD Biosciences). Data were analyzed using FACSDiva or FlowJo v10.7 software (https://www.flowjo.com/solutions/flowjo). Dead cell stain–, EdU-, INS-, and glucagon-labeled (GCG-labeled) cells were detected using the 405, 488, 640, and 561 nm lasers coupled with 525/50, 530/30, 670/14, and 586/15 nm BP filters, respectively. Proliferation was calculated as the fold-change of the percentage of double-positive EdU^+^ and INS^+^ cells over the total INS^+^ cell population, over the control condition. At least 2,300 INS^+^ cells were counted in each sample.

### Insulin secretion in isolated islets.

Following islet isolation from rats at prepuberty (D23–D24), puberty (D37–D38) or young adulthood (D64–D66), islets were incubated in Krebs-Ringer Bicarbonate Buffer (KRBH) (pH 7.4) with 0.1% (w/v) fatty acid-free BSA and 1.0 mM of glucose for 20 minutes. Triplicate batches of 10 islets each were then incubated for an additional 20 minutes in KRBH, 0.1% fatty acid–free BSA, and 1.0 mM glucose, followed by a 1-hour static incubation in KRBH in the presence of 1.0, 8.3, or 16.7 mM glucose. Secreted insulin was measured in the supernatant by RIA. Intracellular insulin content was measured after acid-alcohol extraction.

### 5-HT content in islets.

Following isolation, rat islets were allowed to recover in RPMI-1640 supplemented with 10% (v/v) FBS at 11.1 mM glucose overnight, after which islets were lysed with radioimmunoprecipitation assay (RIPA) buffer (0.5 μL/islet), sonicated, and centrifuged at 12,000*g* for 5 min at 4°C. 5-HT was measured in supernatants using an ELISA kit (LDN). Total protein content was measured using the BCA assay (Thermo Fisher Scientific). 5-HT content was normalized to total protein.

### Quantitative PCR.

Total RNA was extracted from 150 to 200 islets after an overnight recovery in RPMI-1640 supplemented with 10% (v/v) FBS at 11.1 mM glucose using the RNeasy Micro kit (Qiagen). RNA was quantified by spectrophotometry using a NanoDrop 2000 (Invitrogen) and reverse transcribed (1 g). Real-time PCR was performed using the Rotor-Gene SYBR Green PCR kit (Qiagen). Results are expressed as the ratio of target RNA to cyclophilin A (*Ppia*) RNA levels and normalized to control islets. Primer sequences are listed in Supplemental Table 2.

### Statistics.

Data are expressed as mean ± SEM, where *n* represents the number of biological replicates. Statistical analyses were performed using 2-tailed Student’s *t* test or 1- or 2-way ANOVA with Tukey’s, Sidak’s, or Dunnett’s post hoc test adjustment for multiple comparisons, as appropriate, using GraphPad Prism 9 Software. *P* < 0.05 was considered significant.

### Study approval.

All the animal studies were approved by the Institutional Committee for the Protection of Animals at the CRCHUM. Postmortem pancreatic paraffin sections from children 8–15 years old (5 males and 8 females) were provided by the Alberta Diabetes Institute Islet Core and the Pathology Department of Ste-Justine Hospital (Montréal, Quebec, Canada) (Supplemental Table 3). Islets from nondiabetic human donors were provided by the Clinical Islet Laboratory at the University of Alberta (Edmonton, Alberta, Canada) and the National Institute of Diabetes and Digestive and Kidney Diseases–sponsored Integrated Islet Distribution Program (IIDP [RRID:SCR_014387] at City of Hope [Duarte, California, USA], NIH grant no. 2UC4DK098085; Supplemental Table 4). The use of human islets and pancreatic sections was approved by the Institutional Ethics Committee of the Centre Hospitalier de l’Université de Montréal (protocol no. MP-02-2019-7880).

## Author contributions

ALC conceived and designed the study, performed experiments, analyzed data, provided intellectual input, and wrote the manuscript. CG performed experiments and analyzed data. GF performed experiments and analyzed data. ME performed experiments and analyzed data. CT performed experiments and analyzed data. MB performed experiments and analyzed data. DDS provided samples and analyzed data. JG designed the study, provided intellectual input, wrote the manuscript, and supervised the project. VP conceived and designed the study, provided intellectual input, wrote the manuscript, acquired funding, and supervised and administered the project. VP is the guarantor of this work and, as such, had full access to all the data in the study and takes responsibility for the integrity of the data and the accuracy of the data analysis

## Supplementary Material

Supplemental data

## Version 1 09/15/2022

In-Press Preview

## Version 2 11/08/2022

Electronic publication
