# Supplementary material for: β Cell mass expansion during puberty involves serotonin signaling and determines glucose homeostasis in adulthood
Source: JCI Insight. 2022 Nov 8;7(21):e160854. doi: 10.1172/jci.insight.160854 (PMC9675460; doi:10.1172/jci.insight.160854)
Supplement: Supplemental data [file jciinsight-7-160854-s074.pdf]

CASTELL ET AL. - SUPPLEMENTARY MATERIAL

Supplementary Table 1. Sources of antibodies used.

Antibody	Dilution/Concentration	Company	Cat #
**Immunohistochemistry**			
Insulin	1:500	DAKO	A0564
NKX6-1	5µg/ml	DSHB	F55A12
C-peptide	5µg/ml	DSHB	GN-ID4
MKI67	1:500	Abcam	15580
BrdU	1:1000	BD Biosciences	347580
**Flow cytometry**			
Alexa Fluor® Mouse anti-c-peptide	1:25	BD Biosciences	565831
Alexa Fluor® Mouse anti-insulin	1:25	BD Biosciences	565689
PE Mouse Anti-glucagon	1:25	BD Biosciences	565860

Supplementary Table 2. RT-PCR primers.

Target	Forward	Reverse
Ppia	CTTGCTGCAGACATGGTCAAC	GCCATTATGGCGTGTGAAGTC
Ghr	GATGTTCTGAAGGGATGG	GTGGGACTGATGTTGACC
Tph1	TTCTGACCTGGACTTCTGCG	GGGGTCCCCATGTTTGTAGT
Htr2b	AATGTCCTTGGCGGTGGCTGA	GCCAGTGGGAGGGGCCATGTA
Htr1d	TCACGCGGCGGCCATGATTG	CTGCCGCCAGAAGAGCGGTG

Supplementary Table 3. Clinical characteristics of human pancreas donors.

Gender	Age (y)	Weight (kgs)/BMI	Tanner stage (I-V)	Origin
Male	11	35/16.9	I	Ste-Justine
Male	10	31,2/NR	I	Ste-Justine
Male	9	NR/NR	I	Ste-Justine
Male	12	NR/20.80	NR	Alberta
Male	14	NR/21.50	NR	Alberta
Female	8.8	19,9/NR	I	Ste-Justine
Female	14	NR/NR	IV	Ste-Justine
Female	11	NR/NR	II	Ste-Justine
Female	13	55.8/23.2	IV	Ste-Justine
Female	14	NR/NR	IV	Ste-Justine
Female	8	NR/15.90	NR	Alberta
Female	10	NR/16.80	NR	Alberta
Female	15	NR/23.40	NR	Alberta

NR= not reported

Supplementary Table 4. Human islet donors used in study.

**Donors**	1	2	3	4	5	6
Unique identifier	SAMN13972304	SAMN14331402	SAMN15770453	SAMN15877725	SAMN15944113	H2330
Donor Age (years)	49	60	48	31	42	49
Donor Sex (M/F)	M	F	F	M	F	F
Donor BMI (kg/m^2)	34.8	26.2	30.9	27.4	23.5	27,2
Donor HbA1c	5.5	5.1	5.8	5.6	5.4	Not reported
Origin	IIDP	IIDP	IIDP	IIDP	IIDP	Clinical Islet Lab.
Islet isolation center	Southern California Islet Cell Resource Center	The Scharp-Lacy Research Institute	University of Wisconsin	University of Wisconsin	The Scharp-Lacy Research Institute	University of Alberta
Donor history of diabetes?	No	No	No	No	No	No
Cause of death	Cerebrovascular/ stroke	Cerebrovascular/ stroke	Cerebrovascular/ stroke	Head trauma	Cerebrovascular/ stroke	Not reported
Warm ischemia time (h)	Not Reported	Not Reported	Unknown	Not Reported	Not Reported	Not reported
Cold ischemia time (h)	6.3	10.6	5.5	7.8	11.3	Not reported
Estimated purity (%)	90	90	90	95	95	72.5
Estimated viability (%	96	95	98	98	95	85.5
Glucose-stimulated insulin secretion (SI)	SI (G2.8mM-G28mM)= 14.0	SI (G2.8mM-G28mM)= 4.9	SI (G2.8mM-G28mM)= 5.4	SI (G2.8mM-G28mM)= 2.2	SI (G2.8mM-G28mM)= 4.3	Not reported

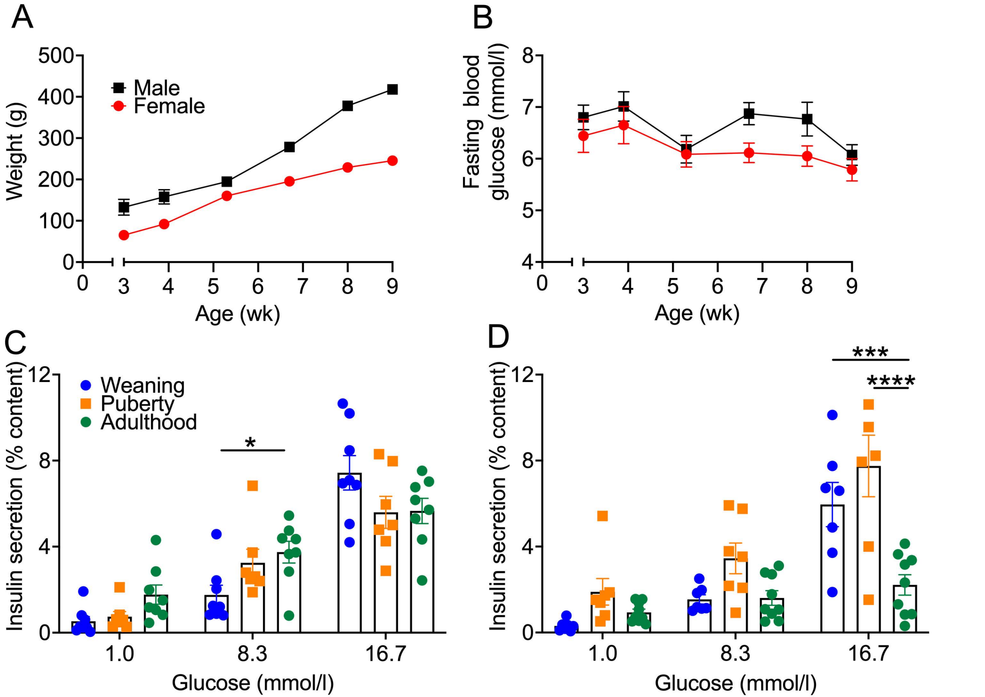

Supplementary Fig. 1. Metabolic parameters and glucose-stimulated insulin secretion in isolated islets in female and male rats from weaning to adulthood. (**A,B**) Body weight (**A**) and fasting blood glucose levels (**B**) in male (black square) and female (red circle) rats from 3-9 wk of age (n=6-9). (**C,D**) Glucose-stimulated insulin secretion presented as a percentage of islet insulin content from islets isolated at weaning (blue circle), puberty (orange square) or adulthood (green circle) in male (**C**) and female rats (**D**) (n=7-9). Data are expressed as mean +/- SEM. *$p<0.05$, ***$p<0.005$, ****$p<0.001$ following two-way ANOVA with Tukey's multiple comparisons test (**C,D**).

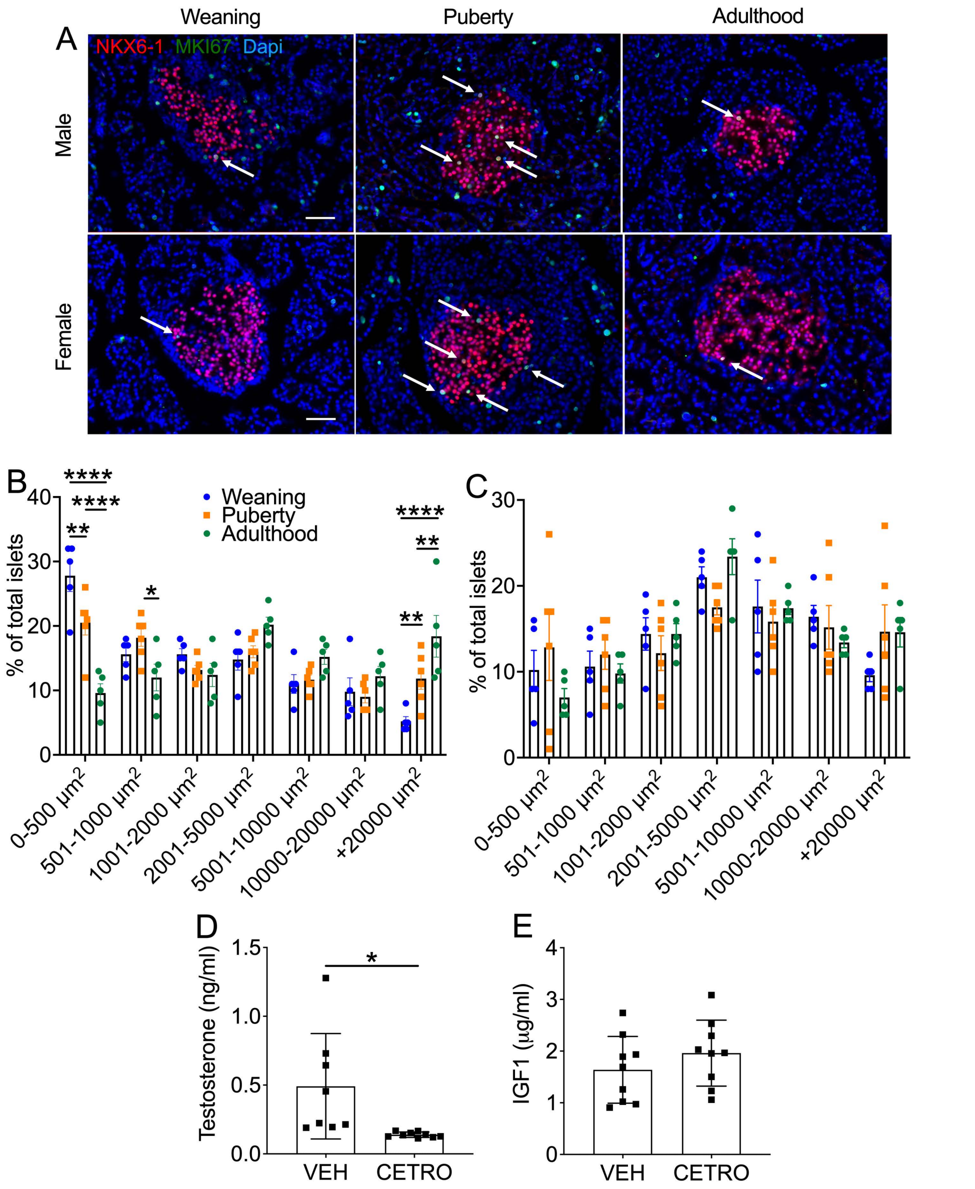

Supplementary Fig. 2. Beta-cell proliferation and islet size in female and male rats from weaning to adulthood and effect of Cetrorelix on hormone levels. (**A**) Representative sections of immunofluorescent staining of pancreatic sections showing NKX6-1 (red), MKI67 (green) and nuclei (Dapi, blue) in male (top) and female (bottom) rats at weaning (3 wk-old), puberty (~5 wk-old) or young adulthood (9 wk-old) (n=6-8). Arrows show positive nuclei for MKI67. Scale bars, 50 µm. (**B,C**) Islet distribution by size in male (n=5-6) (**B**) and female (n=5-6) (**C**) rats at weaning (blue circle), puberty (orange square) or adulthood (green circle) presented as a percentage of total islets. (**D,E**) Plasma testosterone (**D**) and IGF1 (**E**) levels at D38 in rats treated with Cetrorelix (CETRO; 100 µg/d) or vehicle (VEH) from D25 to D37 (n=8-9). Data are expressed as mean +/- SEM. *p<0.05, **p<0.01, ****p<0.001 following two-way ANOVA unpaired with Tukey's multiple comparisons test (**B,C**) or following Student's t-test compared to the VEH group (**D,E**).

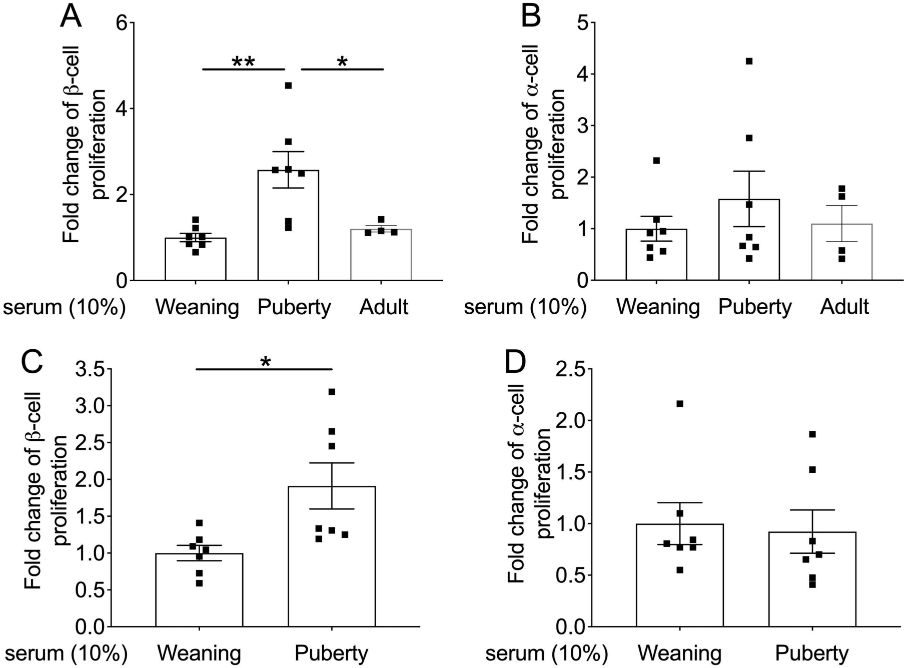

Supplementary Fig. 3. Beta-cell proliferation in response to serum in rat islets isolated at weaning and adulthood. (**A-D**) Male rat islets isolated at weaning (**A,B**) (n=4-7) or adulthood (**C,D**) (n=7) were exposed to pre-pubertal (Weaning), pubertal (Puberty) or adult rat serum for 72h and β- and α-cell proliferation assessed by flow cytometry following staining for EdU and C-peptide (CPEP) or glucagon (GCG), respectively. β- (**A,C**) and α- (**B,D**) cell proliferation presented as the fold-change of the percentage of EdU^+ $CPEP^+$ or EdU^+ GCG^+ cells over $CPEP^+$ or GCG^+ cells, respectively, over the control condition (Weaning serum). Data represent individual values and are expressed as mean +/- SEM. *p<0.05 **p<0.01 following one-way ANOVA with Tukey's multiple comparisons test (**A,B**) or unpaired Student's t-test (**C,D**).

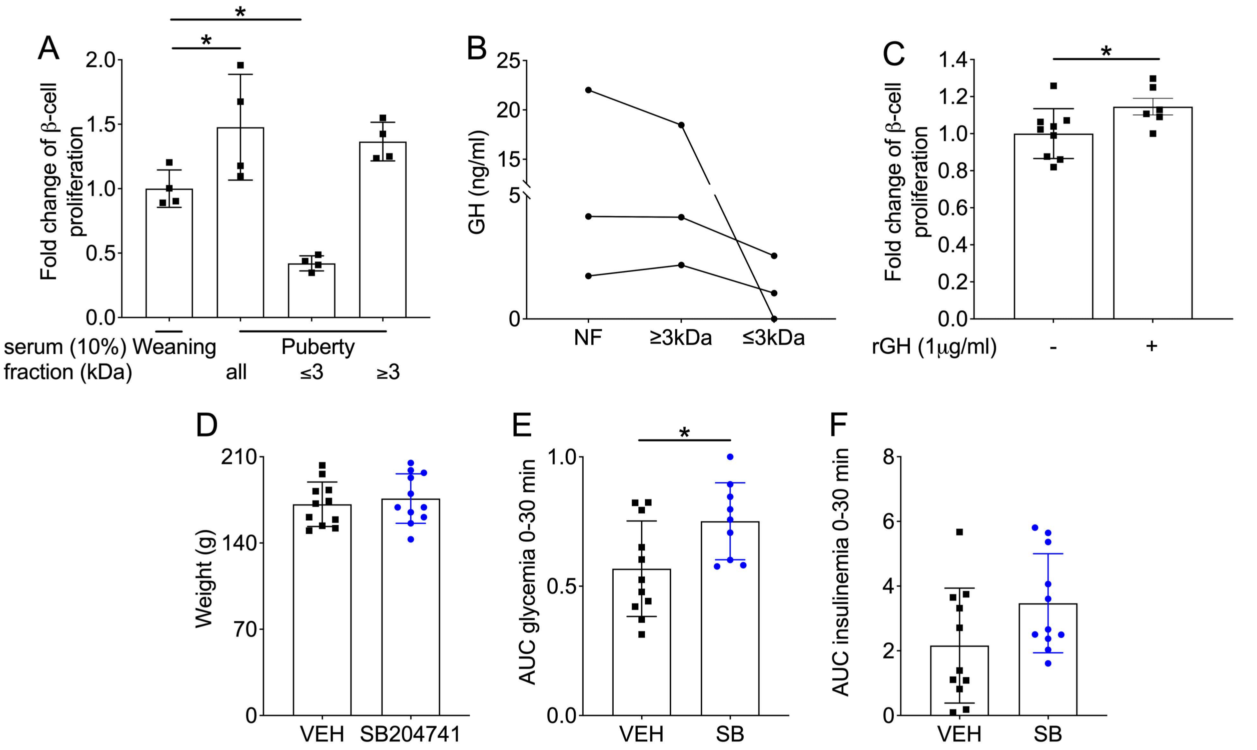

Supplementary Fig. 4. Role of GH and HTR2B signaling in pubertal-induced β-cell proliferation. (**A**) Male rat islets isolated at puberty were exposed to 10% weaning (3 wk-old), pubertal (~5 wk-old) or pubertal rat serum fractions ≤ or ≥ 3 kDa for 72h and β-cell proliferation assessed by flow cytometry following staining for EdU and insulin (INS) and presented as the fold-change of the percentage of EdU^+INS^+ over INS^+ cells over the control condition (Weaning serum) (n=4). (**B**) GH levels in non-fractionated (NF) and fractionated (≤ or ≥ 3 kDa) pubertal rat serum as indicated (n=3). (**C**) β-cell proliferation after 72h exposure to rGH (1 μg/ml) in rat islets isolated at puberty presented as the fold-change of the percentage of EdU^+INS^+ over INS^+ cells over the control condition (n=6-9). (**D-F**) Male rats were i.p. injected with SB204741 (SB, 1 mg/kg/d) (black square) or vehicle (VEH, blue circles) from D25 to D37. Body weight (**D**), AUC of glycemia (**E**) and insulinemia (**F**) after an i.p. dextrose load (1 g/kg) at D38 (n=11). Data are expressed as mean +/- SEM. *p<0.05, following one-way ANOVA with Tukey's multiple comparisons test (**A**) or following Student's t-test (**C-F**).

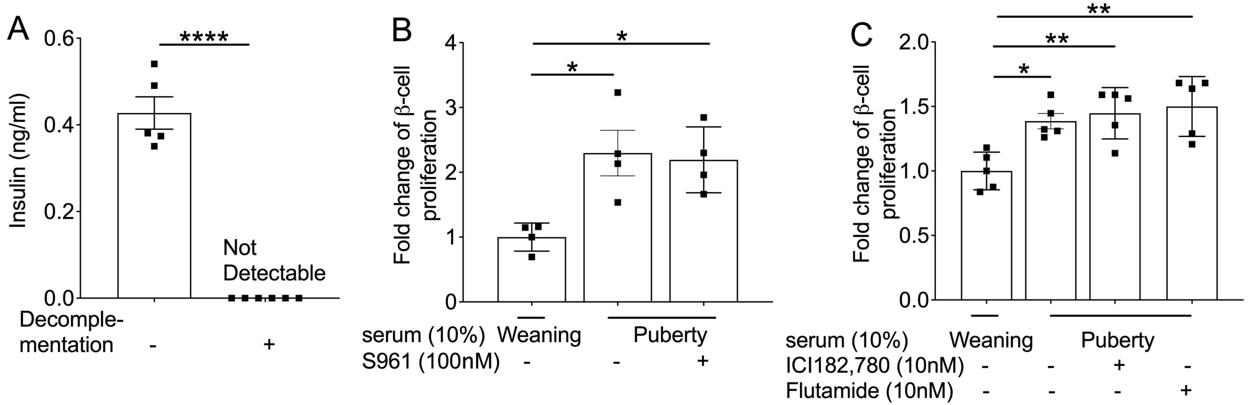

Supplementary Fig. 5. Pubertal serum insulin levels and pubertal serum-induced β-cell proliferation in the presence of insulin, estrogen and androgen receptor antagonists. (**A**) Insulin levels in control and decomplemented pubertal serum (n=6). (**B,C**) Male rat islets isolated at puberty were exposed to 10% weaning (3 wk-old) or pubertal (~5 wk-old) rat serum for 72h in the presence of S961 (100 nM) (**B**) (n=4), ICI182,780 or Flutamide (10 nM) (**C**) (n=5) as indicated and β-cell proliferation assessed by flow cytometry following staining for EdU and insulin (INS) and presented as the fold-change of the percentage of EdU^+INS^+ over INS^+ cells over the control condition (Weaning serum). Data are expressed as mean +/- SEM. *p<0.05, **p<0.01, ****p<0.001, following Student's t-test (**A**) or following one-way ANOVA with Tukey's multiple comparisons test (**B,C**).

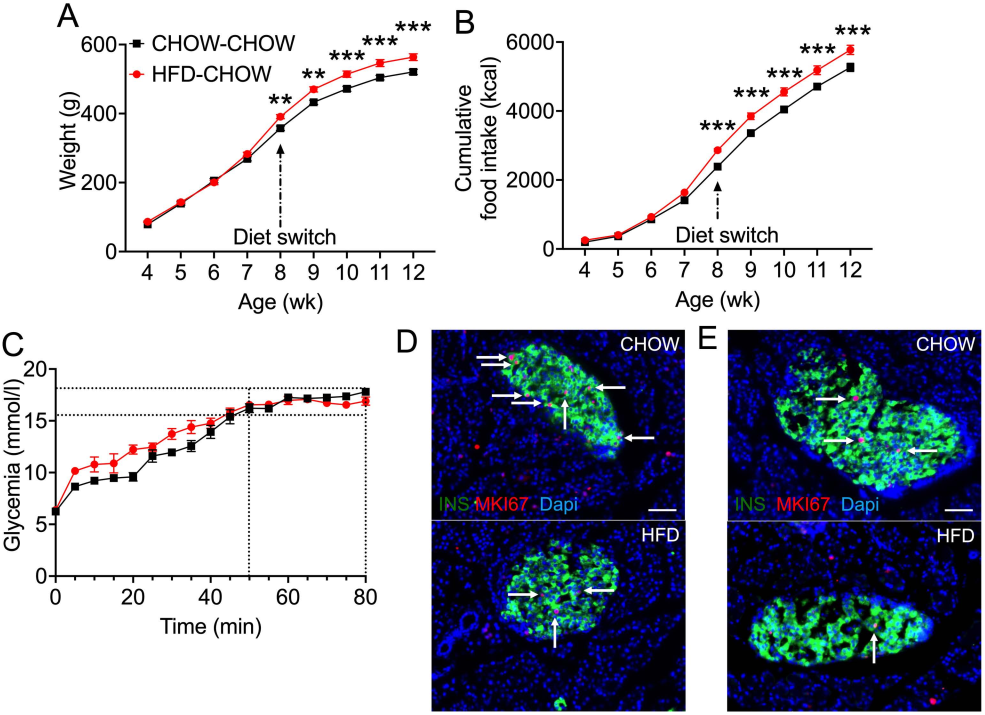

Supplementary Fig. 6. Metabolic parameters in male rats fed a chow or HFD during puberty. Male rats were fed a HFD (HFD, red) or a chow diet (CHOW, black) from 4 to 8 wk of age followed by a switch to a chow diet (HFD-CHOW, red; CHOW-CHOW, black) until 12 wk of age. Body weight (**A**) (n=8-10) and cumulative food intake (**B**) (n=4-5) during the study. (**C**) Glycemia during the HGC in HFD-CHOW and CHOW-CHOW fed animals at 12 wk of age (n=8-9). (**D,E)** β-cell proliferation was assessed at 5 (**D**) (n=3) and 8 (**E**) (n=7-8) wk of age by immunofluorescent staining of pancreatic sections for MKI67 and insulin (INS). Representative sections showing INS (green), MKI67 (red) and nuclei (Dapi, blue). Arrows show positive nuclei for MKI67. Scale bars, 50 μm. Data are expressed as mean +/- SEM. $^{**}p<0.01$, $^{***}p<0.005$, following two-way ANOVA with Sidak's multiple comparisons test (**A,B**).
